# Concomitant EGFR mutation and ALK rearrangement in multifocal lung adenocarcinoma: a case report

**DOI:** 10.1186/s13000-020-00969-1

**Published:** 2020-05-06

**Authors:** Jun Fan, Junhua Wu, Bo Huang, Yili Zhu, Heshui Shi, Xiaofang Dai, Xiu Nie

**Affiliations:** 1 Department of Pathology, Union Hospital, Tongji Medical College, Huazhong University of Science and Technology, 1277 Jiefang Avenue, Wuhan, 430022 Hubei China; 2 Department of Radiology, Union Hospital, Tongji Medical College, Huazhong University of Science and Technology, Wuhan, 430022 China; 3 Cancer Center, Union Hospital, Tongji Medical College, Huazhong University of Science and Technology, 1277 Jiefang Avenue, Wuhan, 430022 Hubei China

**Keywords:** Multifocal lung adenocarcinoma, EGFR/ALK co-altered, Case report

## Abstract

**Background:**

The prevalence of EGFR/ALK co-alterations in patients with NSCLC was low. The several previous studies focused on the simultaneous occurrence of EGFR mutations and ALK rearrangements in a unifocal lung cancer. However, the incidence of multifocal pulmonary adenocarcinomas was increasingly encountered in clinical practice, due to the increased availability and improvement of the thoracic imaging. The clinical relevance of EGFR/ALK co-alterations in multifocal adenocarcinomas required detailed investigation as well.

**Case presentation:**

We present the case of a 57-year-old woman with solid nodule in the left upper lung and a ground glass nodule in the left lower lobe, who underwent radical operation. Pathological examination confirmed multifocal adenocarcinoma, molecular tests revealed that the left upper lung lesion was positive for ALK rearrangement but the left lower lobe displayed EGFR mutation positive separately. The patient pulmonary lesions were well controlled by adjuvant chemotherapy and radiation therapy. When brain metastases occurred, EGFR-TKI was not effective after firstly administration, while subsequent ALK inhibitors were efficient. We retrospective evaluated the oncogenic status of metastatic lymph nodes and found that the driver gene was ALK rearrangement rather than EGFR mutation.

**Conclusions:**

The status of the oncogenic mutations in lymph node metastasis may provide some effective hints for metastasis lesion in other organ or tissue. Therefore, it is recommended to fully evaluate the driver genes in lymph node metastasis after radical resection.

## Introduction

The coexistence of EGFR mutation and ALK rearrangement in patients with lung adenocarcinomas represents a rare molecular subtype of lung cancer [1–3]. There were several previous studies focused on the simultaneous occurrence of ALK rearrangements and EGFR mutations in unifocal lung cancer [4–6]. With the widespread use of low-dose chest computed tomography (CT) and lung cancer screening, the rate of lung carcinoma patients presenting with multiple lesions have been reported from 0.2 to 20%, and most of these cases were multiple pulmonary adenocarcinoma [7]. The prevalence and clinical relevance of EGFR/ALK co-alterations in multifocal adenocarcinomas required detailed investigation as well. Our previous study showed that concomitant EGFR mutation and ALK-rearrangement in synchronous multifocal lung adenocarcinomas was more frequent [8]. Here, we reported a case of patient who diagnosed with multifocal lung adenocarcinomas exhibiting both EGFR mutation and EML4-ALK rearrangement, in order to give some helpful advices for the clinical practice.

## Case presentation

**Fig. 1:**
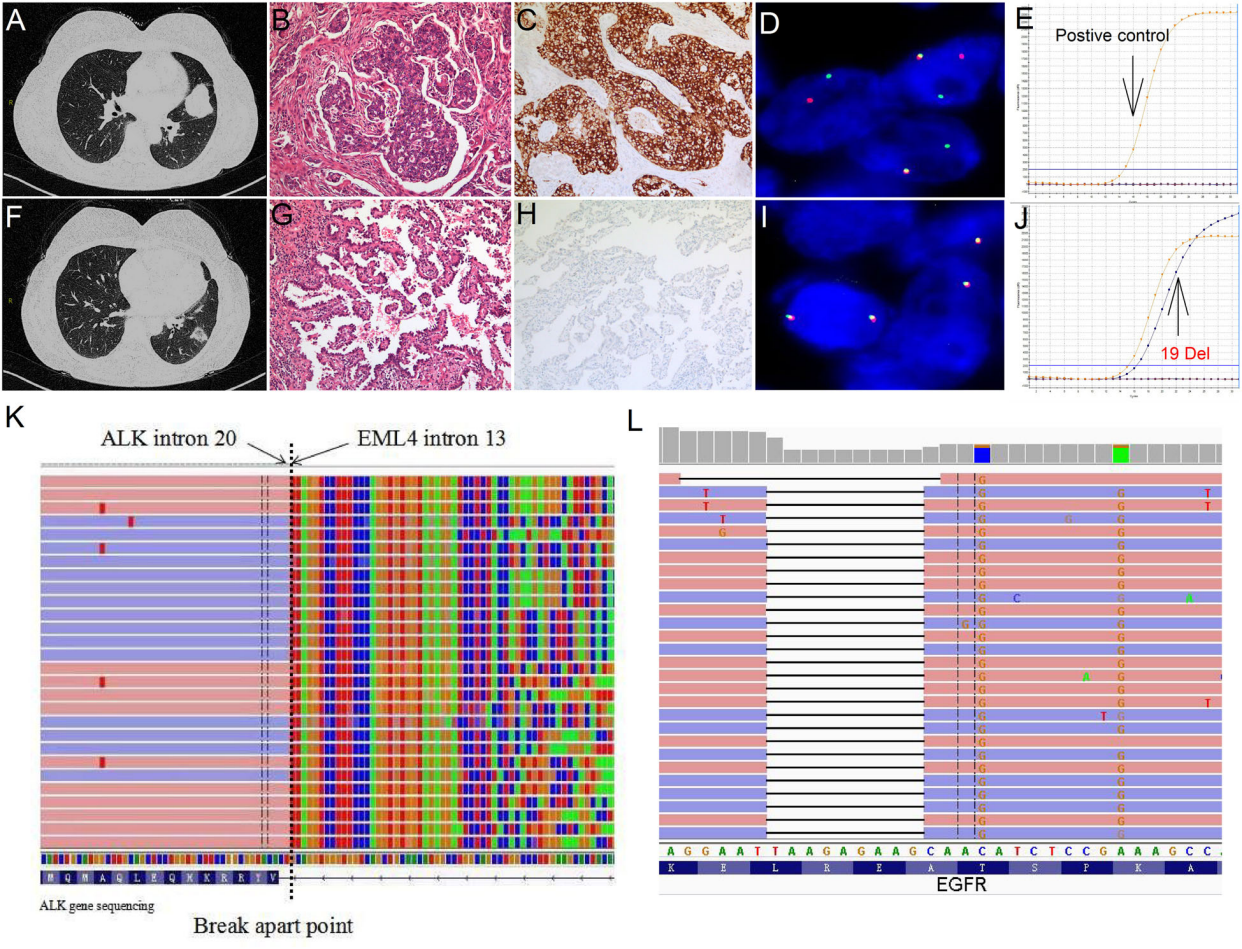
Imaging, pathologic and molecular characteristics of the multifocal adenocarcinoma. Computed tomography showing a solid nodule in the LUL (**a**) and a Ground-Glass Nodule in the LLL(F) . Microscopic showed mixed solid and cribriform patterns in the LUL nodule (**b**) and a Papillary Pattern in the LLL nodule (**g**) (HE; original magnification, × 100). **c** ALK protein was positively expressed in LUL nodule (**c**) but negative for LLL nodule (**h**) (immunohistochemistry; original magnification, × 100). The break-apart fluorescence in situ hybridization assay verified ALK receptor tyrosine kinase gene (ALK) rearrangement in LUL nodule (**d**) but not in LLL nodule (**i**) (FISH; original magnification, × 1000). EGFR mutations were not found in LUL nodule (**e**), but a deletion of EGFR exon 19 presented in LLL nodule (**j**) by the ARMS-PCR method. NGS test confirmed the ALK fusion(E13;A20, relative abundance 6.42%) in LUL nodule (**k**) and EGFR 19 exon deletion (c.2240_2248delTAAGAGAAG, p.L747_A750delinsS, relative abundance 15.58%) in LLL lesion (**l**)

**Fig. 2:**
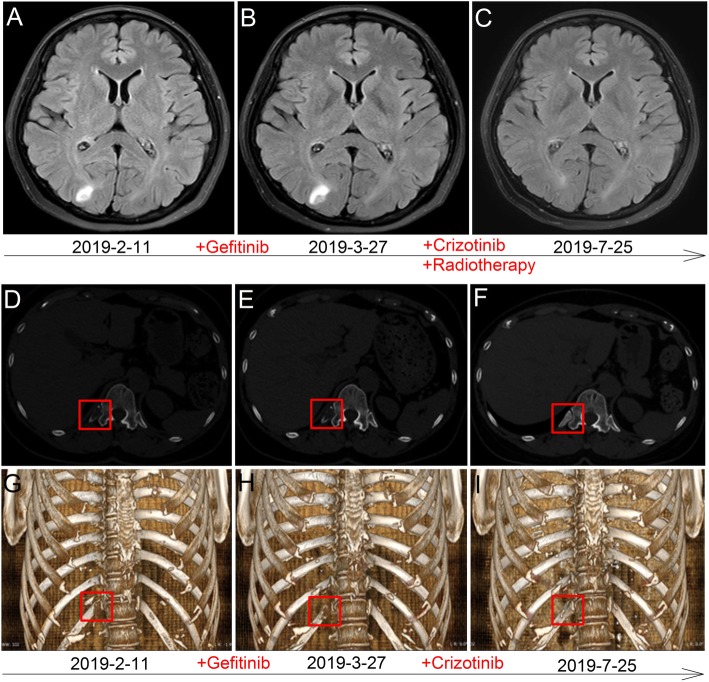
Management of brain and rib metastasis treatment. Head MRI (2019/2/11) showed a 13 mm cranial lesion (upper panel: T2-weighted MRI) (**a**) and bone destruction in the right posterior 12th rib was detected by an CT scan (**d** and **g**). After 1 months of EGFR tyrosine kinase inhibitor treatment, a slight enlargement (to 16 cm) of the brain metastasis lesion (**b**) and bone destruction in 12th rib worsened(**e** and **h**). The patient was treated with crizotinib, brain metastatic lesion received a local tomotherpy contemporary but the rib lesion without radiotherapy. Three months after the therapy, imaging examinations revealed obvious decrease in the size of the brain metastases (**c**). The rib lesion almost disappeared and showed bony restoration (**f** and 2**i**)

**Fig. 3:**
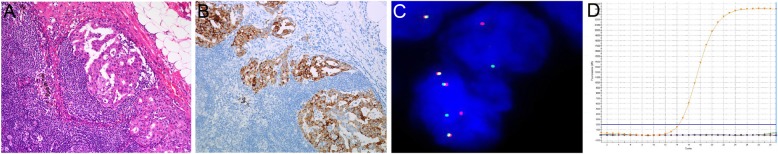
The molecular characteristics in metastatic lymph nodes. Results of H&E Staining (**a**), showed mixed solid and cribriform patterns, Ventana IHC ALK (clone D5F3) (**b**) and ALK gene break apart probe in FISH (**c**), showed positive results, and negative for EGFR mutation by ARMS-PCR analysis (**d**)

A 57-year-old non-smoking woman presented to our hospital with dyspnea in July 2017. Physical examination and laboratory test results showed no significant abnormalities. The chest Computer tomography (CT) scan revealed a 3.2 cm × 3.9 cm solid nodule in the left upper lung (LUL, Fig. 1a) and a 2.3 × 1.6 cm ground glass nodule in the left lower lobe (LLL, Fig. 1f). The patient consequently underwent a LUL resection, a left lower lobe wedge resection, and VATS lymphadenectomy on July 27, 2017. Invasive adenocarcinoma in the LUL lesion was confirmed by the histopathologic examination, with mixed solid and cribriform patterns (Fig. 1b). The results of Immunohistochemistry (Fig. 1c) and fluorescent in situ hybridization (Fig. 1d) revealed that ALK rearrangement was positive. EGFR mutation was not detected by using the amplification refractory mutation system–polymerase chain reaction (ARMS-PCR, Fig. 1e). The LLL nodule also diagnosed as invasive adenocarcinoma, with predominant papillary patterns (Fig. 1g) and EGFR mutation-positive (Fig. 1j), whereas ALK rearrangement was negative (Fig. 1h and i). In addition, the results of 4/7/10/11 lymph nodes pathology confirmed tumor metastasis. NGS–based analysis displayed an ALK rearrangement (EML4 exon 13–ALK exon 20 in LUL nodule, abundance 6.42%, Fig. 1k) and EGFR 19 exon deletion in LLL lesion (c.2240_2248delTAAGAGAAG, p.L747_A750delinsS, abundance 15.58%, Fig. 1l). According to the 8th edition American Joint Committee on Cancer (AJCC) and American College of Chest Physicians Evidence-Based Clinical Practice (ACCP, 3rd edition), the lesions in LUL and LLL were defined as multiple primary adenocarcinomas. Therefore, the tumor classification should be determined separately (LUL: pT2a, LLL: pT1c). Considering the status of lymph node metastasis as N2, the patient was treated as stage 3A non-small cell lung cancer. Initially, the first-line chemotherapy with pemetrexed and nedaplatin (pemetrexed 750 mg/m^2^ with nedaplatin 110 mg/m^2^) was carried out on August 26, 2017. After 6 cycles, the CT scan showed no signs of recurrence and lymphadenectasis. Then the patient received chest radiotherapy with a dose of 54 Gy in 27 fractions. The pulmonary lesions were well controlled. Until February 11, 2019, the patient developed headache, dizziness and thoracodynia. Brain MRI detected a cranial lesion in the right occipital lobe (maximum diameter, 13 mm, Fig. 2a) and CT scan detected a bone destruction in the right posterior 12th rib (Fig. 2d and g), which indicated tumor metastasis. Based on the molecular finding, the patient was delivered the EGFR TKI gefitinib (250 mg daily) at the first. However, the headache and thoracodynia were improved after first month of target therapy. The brain lesion increased to 16 mm (Fig. 2b) and bone destruction worsened (Fig. 2e and h). The therapeutic outcome was considered as progressive disease (PD) and Gefitinib therapy was stopped. As the tumor was positive for ALK rearrangement, the patient was started on crizotinib 250 mg orally twice daily in April 2019. A local tomotherpy was administered to control the brain metastatic lesion contemporary, while no radiotherapy was given to the rib lesion. Three months after the initiation of crizotinib therapy, imaging examinations revealed obvious decreasing of the size of the brain metastases (Fig. 2c). Furthermore, the rib lesion almost disappeared and showed bony restoration (Fig. 2f and i), which was evaluated as partial remission. At the last follow-up (Dec.20, 2019), no evidence of disease progression was found and she still received crizotinib therapy. The treatment of the case suggested that the brain and rib metastasis tumor were both probably driven by ALK gene rather than EGFR. Therefore, we were very interested in the status of the driven gene in the lymph node metastasis of this case. Several tests were performed on metastatic lymph nodes, such as Immunohistochemistry, FISH and ARMS-PCR, and the results showed that all of the metastatic lymph nodes revealed a cribriform growth pattern (Fig. 3a). The pattern suggested that the positive genetic alterations was ALK rearrangement (Fig. 3b and c) rather than EGFR mutation (Fig. 3d). The histomorphology and oncogene alteration of metastatic carcinoma in lymph nodes are consistent with those in the LUL.

## Discussion

Here we report the case presented with one mass at upper lobe and another mass in the lower lobe of left lung. For multifocal lung cancer, the distinction between multiple primaries (MPs) and intrapulmonary metastases (IMs) was of great clinical importance and challenging, as it influenced cancer staging, therapeutic strategy and outcomes [9–11]. After radical surgery, both masses were diagnosed as adenocarcinoma by pathological results, whereas morphological assessment revealed two distinct predominant architectural patterns. In addition, the results of molecular profiling showed that EML4-ALK rearrangement was positive in the LUL, while EGFR gene mutation was detected in the LLL. According to the 8th AJCC staging manual, genomic profiling using comparative genomic hybridization has been suggested to classify tumors effectively but could be hard to implement in routine practice [12]. Sun et al. found that comprehensive histologic assessment combined with a low-grade lepidic component was more accurate clinically and more cost-effective in distinguishing MPs from IMs. In the present case, low-grade lepidic components were identified in both lesions. Tumor in different lobe was classified as MPs according to these factors. Moreover, discrepancy of driver mutations in multiple Lung nodules was evident. From the above, tumors in LUL and LLL was classified as primary tumors and originated from different clones. Accordingly, each tumor should be assigned a separate T, N and M stage as if it were a unifocal lung cancer. In addition, the morphologic and molecular characteristic of the metastases in lymph nodes further supported that lymph node metastatic originated from the LUL lesion, which indicated that the lesion should be diagnosed with pT2aN2M0 and LLL lesion would be determined as cT1cN0M0. The patient received adjuvant chemo- therapy and radiation therapy and pulmonary lesions were well controlled without recurrence. Unfortunately, brain metastasis occurred in February 2019. It is still not frequent enough to define specific guidelines and at present it is not possible to establish the best sequence of treatments. The responses to EGFR and/or ALK inhibitors have been conflicting [4, 13, 14]. Based on the previous molecular finding, the patient was treated the EGFR-TKI. In our case, EGFR-TKI was not effective firstly, while subsequent ALK inhibitors were efficient. Despite the lack of direct evidence, we speculated that brain metastases may be driven by the ALK gene mutation. Our latest study has showed that concomitant EGFR mutation and EML4-ALK rearrangement in lung adenocarcinoma was more frequent in multifocal lesions. Interestingly, there were more lymph node metastasis in patients with multiple ALK gene fusion-positive lesions and more patients with N2 stage tumors. Furthermore, in our retrospective study, metastatic lesions in lymph node were driven by ALK rearrangement rather than EGFR mutation in four cases [6, 8]. All these results suggested that ALK probably played a key role in tumor metastasis of multifocal Lung Adenocarcinoma Harboring EGFR/ALK co-alteration.

The status of the oncogenic mutations in lymph node metastasis may provide some effective hints for metastasis lesion in other organ or tissue. Therefore, it is recommended to fully evaluate the driver genes in lymph node metastasis after radical resection. Beside, we also highlighted the importance of a good morphological approach to multiple lung adenocarcinoma because the phenotypical appearance can, probably, suggest or suspect different oncogenic alterations. Vice versa, even if in the presence of multiple lung adenocarcinoma with the same morphology, is important to test the status of oncogenic mutations in each neoplasia.

Any time, classification of the scarce cases often benefits from multidisciplinary discussion. Furthermore, we need to accumulate more cases to confirm and underlying precise mechanism remains to be clarified.

## Data Availability

The dataset supporting the conclusions of this article is included within the article.

## Abbreviations

EGFR: Epidermal growth factor receptor
ALK: Anaplastic lymphoma kinase
CT: Computed tomography
NGS: Next generation sequencing
TKI: tyrosine kinase inhibitor
IHC: immunohistochemical staining
H&E: Hematoxylin-Eosin
